# A Decellularized Porcine Xenograft-Derived Bone Scaffold for Clinical Use as a Bone Graft Substitute: A Critical Evaluation of Processing and Structure

**DOI:** 10.3390/jfb9030045

**Published:** 2018-07-12

**Authors:** Daniel N. Bracey, Thorsten M. Seyler, Alexander H. Jinnah, Mark O. Lively, Jeffrey S. Willey, Thomas L. Smith, Mark E. Van Dyke, Patrick W. Whitlock

**Affiliations:** 1 Department of Orthopaedic Surgery, Wake Forest School of Medicine, Winston-Salem, NC 27157, USA; ajinnah@wakehealth.edu (A.H.J.); tsmith@wakehealth.edu (T.L.S.); 2 Department of Orthopaedic Surgery, Duke University School of Medicine, Durham, NC 27710, USA; thorsten.seyler@dm.duke.edu; 3 Department of Biochemistry, Wake Forest School of Medicine, Winston-Salem, NC 27157, USA; mlively@wakehealth.edu; 4 Department of Radiation Oncology, Wake Forest School of Medicine Comprehensive Cancer Center, Winston-Salem, NC 27157, USA; jwilley@wakehealth.edu; 5 Department of Biomedical Engineering and Mechanics, Virginia Polytechnic Institute and State University, Blacksburg, VA 24061, USA; mvandyk5@vt.edu; 6 Division of Orthopaedic Surgery, Cincinnati Children’s Hospital Medical Center, Cincinnati, OH 45229, USA; patrick.whitlock@cchmc.org

**Keywords:** xenograft, scaffold, decellularized, osteoconductive, bone graft, porcine

## Abstract

Background: Bone grafts are used in approximately one half of all musculoskeletal surgeries. Autograft bone is the historic gold standard but is limited in supply and its harvest imparts significant morbidity to the patient. Alternative sources of bone graft include allografts, synthetics and, less commonly, xenografts which are taken from animal species. Xenografts are available in unlimited supply from healthy animal donors with controlled biology, avoiding the risk of human disease transmission, and may satisfy current demand for bone graft products. Methods: In the current study, cancellous bone was harvested from porcine femurs and subjected to a novel decellularization protocol to derive a bone scaffold. Results: The scaffold was devoid of donor cellular material on histology and DNA sampling (*p* < 0.01). Microarchitectural properties important for osteoconductive potential were preserved after decellularization as shown by high resolution imaging modalities. Proteomics data demonstrated similar profiles when comparing the porcine bone scaffold against commercially available human demineralized bone matrix approved for clinical use. Conclusion: We are unaware of any porcine-derived bone graft products currently used in orthopaedic surgery practice. Results from the current study suggest that porcine-derived bone scaffolds warrant further consideration to serve as a potential bone graft substitute.

## 1. Introduction

A bone scaffold is a three dimensional matrix used to fill bone voids and support the body’s intrinsic regenerative potential [1]. As osteogenic cells migrate and adhere to the scaffold, new bone is formed and the scaffold is eventually degraded and turned over [2]. The ideal bone graft substitute should mirror the properties of the “gold standard” autograft, and thus, be biocompatible, osteoconductive, osteoinductive, osteogenic, and also be readily available without risk of disease transmission [3]. Scaffolds can be constructed from synthetic materials that are sterile and non-immunogenic, allowing them to be safely implanted in a host without risk of disease transmission and minimal risk of an inflammatory reaction. However, to more closely approximate the morphology and biomechanical properties of autograft bone, scaffolds can also be derived from natural bone to optimize integration into surrounding host tissue [4]. Perhaps the most widely utilized bone scaffold is demineralized bone matrix (DBM), which is derived from human allograft bone. DBM serves as an osteoconductive matrix with osteoinductive potential imparted by the retained growth factors from the donor [5]. Biologic activity of DBM depends on donor health and bone processing techniques which ultimately results in considerable variability between DBM products [6]. The cost of one milliliter of DBM often exceeds $100, making their application cost prohibitive in the treatment of larger osseous defects. Scaffolds derived from alternative sources of natural bone, such as xenograft bone (non-human), could afford the same osteoconductive benefits as DBM at a lower cost, avoid the risk of human disease transmission and be immediately available in relatively unlimited quantities from a larger pool of healthy donors with controlled biology [7]. If a xenograft-derived scaffold also retained its donor’s osteoinductive growth factors, the product could potentially serve the same clinical purpose as DBM.

Bovine bone has previously been transplanted into human patients, but orthopaedic clinical series showed poor results with frequent graft rejection and failure to integrate within host tissue [8,9,10,11,12,13]. Very few orthopaedic studies have considered bone graft substitutes derived from porcine xenograft sources [14,15,16,17,18,19,20,21]. To the best of the authors’ knowledge, there are currently no commercially available porcine-derived bone graft products available for clinical use in orthopaedic surgery and there are no clinical reports of porcine bone grafting into human subjects in the orthopaedic literature. Porcine species share similar anatomy, physiology, and genetic makeup with human species [14,17] and historically have served as excellent xenograft donors to human recipients [22,23,24]. While orthopaedic literature has limited reports of xenograft use, facial reconstruction and periodontal literature has more experience with these products [25,26,27,28,29]. The biology of oral and facial grafting sites differs from sites of orthopaedic pathology treated with bone grafting but it is still important to recognize the recent successes seen with xenografts and various bone graft substitutes used in treatment of facial and periodontal pathology [30,31,32] with improved patient outcomes and quality of life [25,26]. This literature supports further consideration in orthopaedic applications as well.

A novel decellularization and chemical oxidation protocol developed in our laboratory has previously derived tendon, meniscus, and ligament scaffolds from both human and animal tissue [33,34,35,36,37]. Results from these studies showed that the decellularization technology can derive biocompatible and pathogen-free tissue scaffolds. We proposed that this protocol could be used to decellularize bone to derive scaffolds that could potentially serve as bone graft substitutes for clinical use. We hypothesize that cancellous bone in porcine femurs can be decellularized to produce a scaffold that: (1) is devoid of donor cellular material; (2) maintains the key ultrastructural properties of native bone critical for osteoconductive potential; (3) maintains similar biomechanical properties to donor bone; and (4) has a protein composition similar to DBM, an analogous human-derived bone graft product currently in clinical use with proven osteoinductive potential [5,38,39].

## 2. Results

### 2.1. Structural Imaging

#### 2.1.1. Macroscopic Field Images

**Figure 1:**
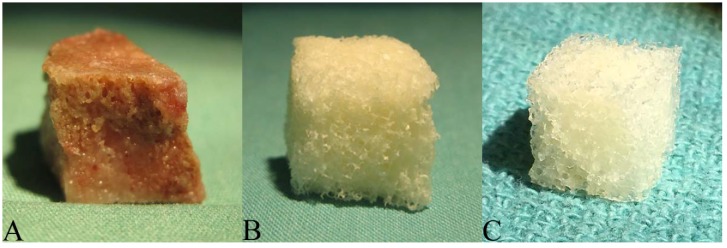
Macroscopic Field Images: (**A**) Donor cancellous bone harvested from the distal metaphysis of porcine femurs; (**B**) Bone scaffold derived from donor specimens using a patented decellularization and oxidation protocol that combines physical and chemical processing treatments; (**C**) Commercial Demineralized Bone Matrix (CONFORM^®^ FLEX Demineralized Cancellous Bone, DePuy Synthes, Distributed by Musculoskeletal Transplant Foundation).

Donor porcine bone (Figure 1A) post-oxidation was devoid of adipose marrow contents and took on a bright white, uniform appearance. Post-lyophilization, the scaffolds (Figure 1B) became more brittle with obvious reduction in density. Decellularized scaffolds were similar in appearance and texture to demineralized bone matrix (DBM) (Figure 1C).

#### 2.1.2. Scanning Electron Microscopy

**Figure 2:**
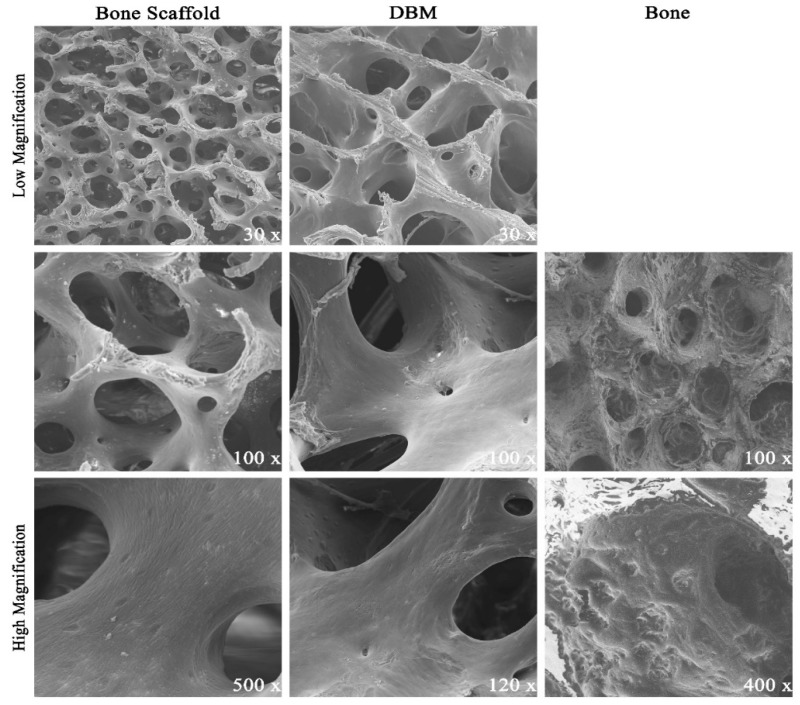
Comparative Scanning Electron Micrographs: Donor porcine cancellous bone (“Bone”) was decellularized to derived bone scaffolds. Representative micrographs appreciate the porous surface architecture. Demineralized Bone Matrix (DBM) is a commercially available human cancellous bone graft product. DBM pores appeared larger but high magnification surface was similar. Bone appeared less porous with marrow contents filling pores.

Scanning electron micrographs highlighted the porous surface architecture present on both the donor bone and scaffold (Figure 2). Overall microarchitecture of scaffolds was markedly similar to unprocessed donor bone, suggesting that the decellularization protocol did not grossly compromise the osteoconductive ultrastructure. Blade etchings from commercial production were visible on DBM surfaces (Figure 2, DBM 30× magnification). Pores on DBM samples were notably larger than those on porcine bone and scaffold specimens.

#### 2.1.3. Micro-CT Imaging

**Figure 3:**
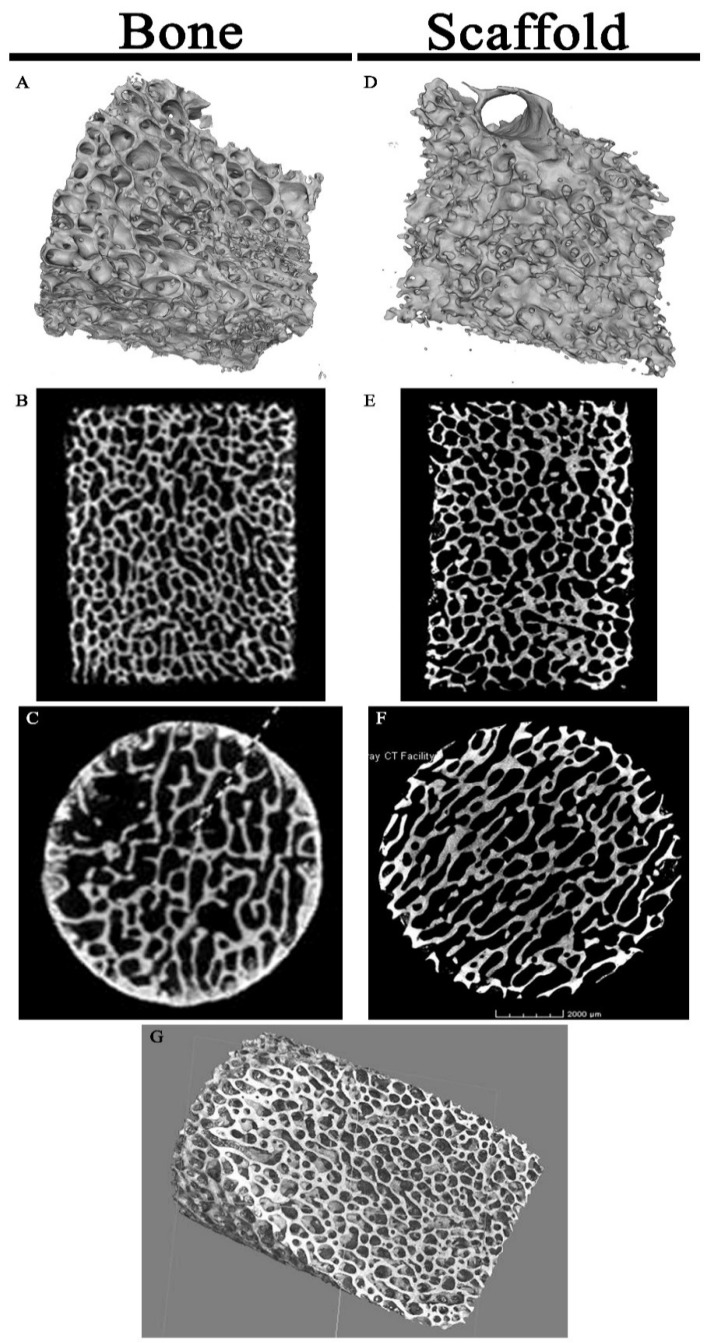
Micro-CT Imaging of Specimens: Porcine cancellous bone micrographs (**A**–**C**) and scaffold micrographs (**D**–**F**) were similar in appearance on 3-dimensional projections (**A**,**D**), coronal (**B**,**E**), and axial (**C**,**F**) cuts. (**G**) High resolution scaffold 3-dimensional projection through the matrix interior displays the highly porous architecture.

Micro-CT imaging showed similar results for both the porcine bone and scaffold specimens with no change in gross architecture (Figure 3). The decellularization protocol uses a weak acid (peracetic acid) but this did not demineralize the substrate enough to yield changes detectable by micro-CT imaging.

### 2.2. Assessment of Decellularization

#### 2.2.1. Histology

**Figure 4:**
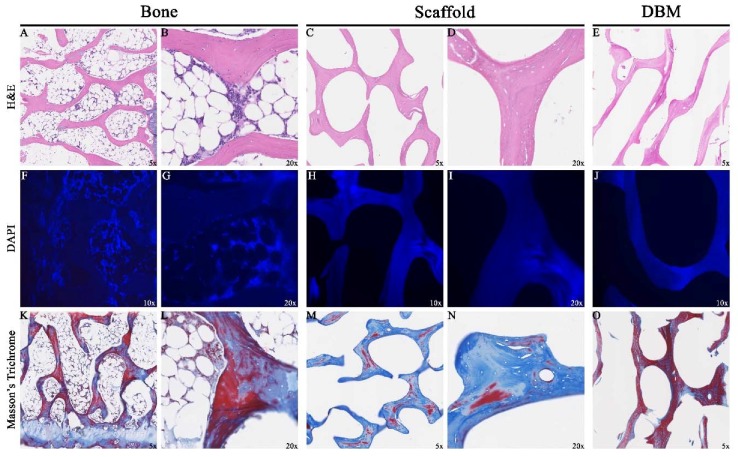
Assessment of Decellularization by Histology: Representative sections 5 μm thick taken from specimen midsections showed removal of cellular contents (**A**,**B**) in the scaffolds (**C**,**D**). 4′,6-diamidino-2-phenylindol (DAPI) sections stained abundant cell specific material (**F**,**G**) which was not seen in scaffolds (**H**,**I**). Collagen content distribution was highly variable in specimens (**K**–**N**). Demineralized Bone Matrix (DBM) sections (**E**,**J**,**O**) were similar in appearance to the scaffold.

H&E stained sections showed that marrow contents (Figure 4A,B) and osteocytes within lacunae of donor bone were removed after decellularization (Figure 4C,D). Consistent with these findings, 4′,6-diamidino-2-phenylindol (DAPI) stained sections showed an abundance of nuclear material in the source bone (Figure 4F,G) that was removed after decellularization (Figure 4H,I). DBM sections (Figure 4E,J,O) were similar in appearance to scaffold sections.

#### 2.2.2. Measurement of DNA Content

DNA concentration in donor bone was 14.52 ± 7.7 μg/mL and 0.244 ± 0.09 μg/mL in scaffolds (*p =* 0.001), representing a 98% reduction in DNA content achieved by the decellularization protocol.

### 2.3. Structural Characterization

#### 2.3.1. Ultrastructure Measurements

The density of donor bone samples (Table 1) was significantly greater than decellularized scaffold samples (Bone density 1366 ± 19.82 mg/mL, Scaffold density 570.4 ± 91.32 mg/mL, *p* < 0.0001). Porosity was slightly greater in scaffolds (79.5 ± 9.1%) than donor bone (69.1 ± 11.1%) but this difference was insignificant (*p* = 0.20). Pore size was also similar (*p* = 0.77) between scaffolds (474.2 ± 76.2 µm) and bone specimens (458.5 ± 66.3 µm). Thickness of the bone struts in the trabecular architecture as well as anisotropy were not different between donor bone and scaffold specimens (Table 1).

#### 2.3.2. Finite Element Analysis (FEA)

Finite element analysis (FEA) of bone and scaffold specimens (Table 2) showed no significant differences in yield stress (von Mises), stiffness, or failure loads for the subvolumes of samples analyzed. There was significant variability in scaffold samples however (Table 1).

#### 2.3.3. Mechanical Compression Testing

Mechanical compression testing results supported the trends shown in the FEA analysis (Table 2). Significant differences were found between bone and scaffold specimens for maximum strain at failure (*p* < 0.0001), stiffness (*p* < 0.0001), and the elastic modulus (Young’s modulus *p* < 0.0001). Load at failure was not different between scaffold and bone specimens.

### 2.4. Proteomics

Protein composition identified by mass spectrometry analysis of porcine bone scaffold and human DBM samples were similar (Table 3). A variety of structural proteins serving to support the extracellular matrix as well as proteins providing particular biologic functions were identified. Proteins supporting a variety of intra and extracellular signaling pathways (Chondroadherin, Lumican, Biglycan) were found in nearly all porcine and human specimens. Proteins found in blood (Annexin A5, Hemoglobin subunit beta) were identified in more pig than human specimens likely due to differences in specimen preparation and processing techniques between our methods and those of the commercial vendor.

## 3. Discussion

Our decellularization protocol effectively removed porcine cellular material, supported by the 98% reduction in DNA content observed. Removal of donor cellular material is critical in preventing host mediated macrophage response after transplantation [41]. In previous studies applying the same decellularization protocol to different soft tissues, we recorded a 67% reduction in DNA content from avian FDP tendons [37], a 55% reduction from ovine menisci [34], and a 61% reduction from porcine patellar tendons [33]. The relatively increased porosity of cancellous bone likely permitted deeper penetration of the treatment chemicals and ultimately resulted in more efficient decellularization in comparison to the other less porous soft tissues previously treated. Comparative histology of commercial DBM specimens was similar in appearance and also devoid of cellular material on DAPI sections. Decellularization did not appear to alter the micro-architecture of the bone scaffold critical for preservation of osteoconductive potential.

Density of porcine cancellous bone taken from the distal femoral metaphysis was ~1.4 g per cubic centimeter, which is slightly greater than reports of human cancellous bone harvested from the calcaneus (1.135 g/cm^3^) or iliac crest (1.098 g/cm^3^) [4]. While decellularization did significantly reduce density of processed specimens, it did not significantly alter the micro-architecture of the donor bone. The significance of micro-architecture to osteoconductivity is well established [42], and bone graft substitutes should generally seek to mimic native bone architecture [43,44,45,46,47]. Of critical importance are the porosity and pore size of scaffolds, which affect efficiency of cell seeding, diffusion into the scaffold, and the mechanical strength of the scaffold [48]. Highly porous structures permit higher mass transport, vascularization, osteo-integration from adjacent bone, and infiltration of both osteoblasts and osteoclasts to mediate scaffold remodeling [49,50]. Less porous structures are stronger and have greater surface area for protein adherence and cell attachment, but with less porosity this is limited to the periphery of the structure and ultimately limits ingrowth and cell penetration [43,49,50]. Porosity of human cortical bone is 3–12% while porosity of cancellous trabecular bone is highly variable ranging from 50% to 90% depending on location and donor biology [4,42]. We saw a range of porosity in our porcine donor specimens that was similar to this human data. Decellularized scaffold porosity was 80% on average. This is comparable to the reported porosity of DBM which ranges from 62% to 88% [51,52]. Objective parameters for pore size are debated in the tissue engineering literature with a pore diameter of 100–500 μm considered ideal [43,53]. Pore diameter greater than 40 μm is required for osseous ingrowth, and osteoid formation requires a minimum pore diameter of 100 μm [4,43]. Pore size was highly variable in our specimens typically falling between 400 and 600 μm, which are defined as large pores [48,49]. By comparison, DBM pore size averages 200–500 μm [54], but is highly variable when derived from cancellous bone. Limited data on porcine demineralized cancellous bone is available, however, one study [55] reported findings similar to ours with 62% porosity and 520 μm average pore size in specimens derived from the proximal metaphysis of porcine femurs.

Similar to porosity, the biomechanical properties of cancellous bone are also highly variable and depend on bone location, as well as a number of donor variables such as specie, gender, and age [4,56,57,58]. Young’s modulus of human cancellous bone ranges from 50 to 389 MPa [16,59], with some reports in the GPa range. This depends on the orientation of specimen loading owing to the effects of anisotropy [42]. Similar to human cancellous bone, we found porcine cancellous bone average modulus in the range of 200–300 MPa. After decellularization, we consistently found that bone scaffolds were less stiff and had greater deformation at failure. Given that matrix density is directly proportional to its modulus [56,60,61], this result was partially expected knowing that bone specimens were significantly more dense than scaffold specimens. Chemical modifications made to the bone during decellularization also explain the reduction in elastic modulus. Although peracetic acid (PAA) is a weak acid, it does demineralize bone to a small extent which softens the structure, makes it tougher, and decreases elastic modulus [62]. Lyophilization of tissues has also been shown to compromise mechanical strength [62,63,64] and may have altered the elastic modulus in the final stage of scaffold production. When lyophilized specimens are rehydrated, as we did before destructive mechanical testing, they typically lose 20% stiffness and 18.9% ultimate strength [63]. Rehydration of specimens more closely mimics physiologic conditions, however, and was appropriate for testing.

Scaffolds derived from cancellous bone blocks are not intended to serve as load-bearing structures. However, the biomechanical properties of the structure still bear clinical significance in supporting bone regeneration. Bone grafts and scaffolds should mimic the fundamental mechanical properties of bone and closely match the surrounding tissue’s compressive strength, toughness, and stiffness as closely as possible [65,66]. The scaffold cannot support large physiologic loads, but it must maintain its structure long enough to preserve the void it fills until remodeled by host ingrowth [65]. Without appropriate stiffness and strength at the implantation site, the scaffold may be resorbed by the host which will prevent progressive bone formation, remodeling, and osseous union with host ingrowth [66]. Stiffness of the scaffold has also been shown to direct stem cell fate with softer matrices directing cells to neurogenic lineages and stiffer matrices mimicking the cross-linked collagen structure of osteoid that pushes cells towards osteogenic lineages [67,68,69,70]. As the cells pull on the matrix through focal adhesions, they gauge the matrix’s stiffness by the amount of resistance they sense [67]. Stiff matrices are defined by elastic moduli greater than 25 kPa and can have osteoinductive effects on stem cells [68]. Although significantly lower than donor bone, the modulus of our scaffold was on the order of MPa, thus making it a stiff matrix capable of differentiating stem cells down osteogenic pathways. The above biomechanical indices are likely affected by anatomic location of the donor bone. We harvested bone from the distal metaphysis of femurs because they are weight bearing bones we found to yield the largest quantity of cancellous bone for processing.

Very few studies use mass spectrometry to analyze protein composition of human DBM [71,72,73], and to our knowledge, no study has compared protein profiles of a xenograft derived bone scaffold against human DBM. The protein composition of the structural and biologic elements of our scaffold was remarkably similar to human DBM, a clinical product with proven osteoconductive and osteoinductive potential. Despite deriving our scaffold from a non-human species, this data is critical in demonstrating the structural and functional similarity of porcine and human-derived bone scaffolds, suggesting they may serve similar biologic as well as structural roles in bone grafting.

Several limitations exist in the current study. We were unable to control some porcine donor variables such as potential comorbidities, age, and specimen contamination at time of acquisition. All donor bone was harvested from the distal metaphysis of femurs, but we did not control for the variable density of the cancellous bone which has previously been shown to affect cell attachment and differentiation [52]. While histology showed convincing evidence of decellularization and DNA quantification supported this finding, it is unclear if DNA reduction is the most accurate assessment of effective decellularization. Cellular remnants and extracellular proteins not measured by DNA quantification may be as immunogenic as whole cells [41]. The extent of bone demineralization caused by peracetic acid treatment in our protocol was not defined in the current study. The biomechanical consequences of this cannot be distinguished from the effects of lyophilization or potential degradation of the organic matrix. Lastly, proteomics data is limited by the unknown biologic activity of detected proteins. Mass spectrometry identifies proteins by primary structure alone and does not characterize secondary, tertiary, or quarternary protein structures which are critical to protein function and biologic activity. Despite preserving proteins believed to impart osteoinductive potential, we cannot conclude that these porcine proteins will impart the same effects on human cells. Further in vivo study will be required to determine if our xenograft will yield clinical translation and those animal studies have not been performed. We can justify further in vivo study based on some of the results seen in the facial reconstruction and periodontal literature [25,26,28,29,31,74,75,76,77]. A recent review by Cicciu et al. [26] highlighted the clinical and animal models in facial bone reconstruction using various xenograft derivatives with particular attention to marine origins of bone graft substitutes. A non-human primate study by Herford et al. [75] treated mandible defects with a combination of bovine bone and showed that addition of recombinant human BMP-2 increased bone and soft tissue regeneration with distraction osteogenesis. The additive effects of BMP are another possibility to explore with our porcine xenograft given the positive results seen in other animal models. In vivo animal model study with our porcine xenograft will be the next essential step in study of its osteoinductive potential and potential use in clinical practice.

Results presented in the current study show that our decellularization protocol can derive a scaffold from porcine bone that is devoid of donor cellular material while preserving the microarchitecture critical to osteoconduction, and, potentially, the growth factors thought to contribute to the osteoinductive potential of bone graft substitutes. While the overall structural parameters and yield strength were preserved after decellularization, the protocol did alter the biomechanical properties of the starting material as seen by significant decreases in bone stiffness.

## 4. Materials and Methods

### 4.1. Bone Scaffold Production

#### 4.1.1. Acquisition of Animal Tissue

The femurs from freshly sacrificed female pigs (*Sus scrofa domesticus)*, aged 3–4 years old, weighing 450–650 lbs were obtained from City Packing Company (Burlington, NC, USA) with an educational and research permit granted by the North Carolina Department of Agriculture and Consumer Services. Pigs are commonly slaughtered at this age, therefore, we elected to study pigs of this age to be representative of the potential porcine donor pool. Femurs were transported to our laboratory on ice and immediately stripped of soft tissue before being frozen at −20 °C for later use.

#### 4.1.2. Physical Processing of Specimens

Cross sections 6–10 mm thick were taken from the distal end of femurs using an upright stainless-steel autopsy grade band saw (Band Saw K220, KUGEL Medical, Regensburg, Germany). Only sections taken from the metaphysis were kept for processing, while specimens containing articular and subchondral elements were discarded. Sections were mounted to a drill press (DELTA Power Equipment Corporation, Milwaukee, WI, USA) and bone plugs were machined with a 3/8” plug cutter (Harbor Freight Tools, Calabasas, CA, USA). To prevent thermal necrosis of specimens, the bone sections were drilled while submerged in ice-chilled normal saline. Bone plugs were then washed at high pressure to flush out marrow contents.

#### 4.1.3. Decellularization and Chemical Oxidation Protocol

Specimens were processed as previously described [37] using a patented decellularization and oxidation protocol [78]. Briefly, cancellous bone plugs were transferred into autoclaved screw-top glass flasks (Corning Inc., Corning, NY, USA) containing 1000 mL of DNase/RNase-free, distilled water (Gibco, Grand Island, NY, USA). Flasks were placed on a rotating shaker (Barnstead MaxQ400, Dubuque, IA, USA) at 200 rpm, 37 °C, for 72 h with fresh water exchanges every 12 h. Water was discarded and 500 mL of 0.05% trypsin-EDTA (Gibco, Grand Island, NY, USA) was added to flasks before returning them to the rotating shaker. After 2 h, the trypsin solution was discarded and 500 mL of Dulbecco’s Modified Eagle’s Medium (DMEM) high-glucose (Gibco, Grand Island, NY, USA) containing 10% fetal bovine serum (FBS) (Valley Labs, Winchester, VA, USA) and 100 I.U./mL Penicillin, 100 μg/mL Streptomycin, 0.25 μg/mL Amphotercin B (Gibco, Grand Island, NY, USA) was added to halt trypsin digestion and maintain an aseptic preparation. Flasks were returned to the shaker for 24 h before discarding the DMEM solution, adding 1000 mL of fresh DNasefree/RNase-free distilled water and returning it to the shaker for 24 h. Water was discarded, and a 1000 mL solution of 1.5% Peracetic Acid (Sigma, St. Louis, MO, USA), and 2.0% Triton X-100 (Sigma) prepared in distilled, deionized water was added, and flasks were returned to the rotary shaker for 3 h. The oxidizing solution was discarded and residual peracetic acid was removed by flushing the samples with 1000 mL autoclaved, deionized water and then repeated washes in 1–3 h cycles (each at 200 rpm, 37 °C). Wash cycles were repeated until peracetic acid was no longer detectable by peracetic acid test strips (Sigma). Specimens were then transferred into conical tubes, frozen at −80 °C for 24 h, lyophilized (LabConco, Freeze Dry System, Kansas City, MO, USA) for 24 h, and stored at −80 °C until further use.

### 4.2. Structural Imaging

#### 4.2.1. Scanning Electron Microscopy

Donor porcine bone, scaffolds, and DBM samples were mounted on aluminum stubs (Ted Pella, Redding, CA, USA) with carbon tape and gold sputter coated at 30 mTorr. Specimens were imaged on a Hitachi S-2600 scanning electron microscope (Hitachi, Tokyo, Japan).

#### 4.2.2. Micro Computed Tomography

Scaffold and bone specimens were scanned in cross section at 10 μm voxel resolution and reconstructed into 3d images on a Scanco 80 micro-CT system (Scanco USA, Wayne, PA, USA). Images were inverted to visualize the porous domains so pore size and morphology could be quantified using methods previously described by Lin et al. [59]. Additional specimens were scanned on a nanotom micro-CT (phoenix nanotom^®^ m, General Electric, Fairfield, CT, USA) for high resolution images.

### 4.3. Assessment of Decellularization

#### 4.3.1. Histology

Bone and scaffold specimens were fixed in 10% neutral buffered formalin (C) for 48 h, washed with deionized, distilled water, and decalcified for 48–72 h with 20× volume of Immunocal^®^ (Decal Chemical Corp, Tallman, NY, USA). Specimens were then transferred into Cal-Arrest (Decal Chemical Corp) for 2–3 min before being stored in 70% ethanol. Specimens were then processed and embedded in paraffin. Sections 5 µm thick were taken from the midportion of samples on a microtome, mounted, and stained with hematoxylin and eosin (H&E) (Sigma), Masson’s trichrome (Sigma) or 4′,6-diamidino-2-phenylindole (DAPI) mounting media (ProLong^®^ Gold Antifade Mountant, Thermo Scientific, Waltham, MA, USA). Representative light micrographs were captured with the Olympus VS-110 Virtual Imaging System (Olympus, Center Valley, PA, USA) and fluorescent micrographs with a Zeiss Axioplan2 system (Carl Zeiss, Oberkochen, Germany).

#### 4.3.2. DNA Quantification

DNA content was measured in the donor cancellous bone (*n* = 6 femurs) and decellularized scaffold to assess efficacy of decellularization. DNA content was normalized to specimen volume to correct for changes in specimen density after decellularization. Volume was calculated by measuring the height and diameter of specimen plugs with a jeweler’s caliper (Esslinger, St. Paul, MN, USA). Samples were individually cryomilled (6870 Freezer/Mill^®^, Thomas Scientific, Swedesboro, NJ, USA) in liquid nitrogen, and lysed in 3× volume of mammalian protein extraction reagent (M-PER^®^, Thermo Scientific, Rockford, IL, USA) with protease inhibitors (cOmplete ULTRA Tablets, Roche, Basel, Switzerland). DNA concentration of lysates was measured with Quant-iT™ PicoGreen^®^ dsDNA Assay Kit (Thermo Scientific) in polystyrene plates (Corning^®^ 96-well) on a fluorescence microplate reader (FLUOstar Optima Plate Reader, BMG Labtech, Ortenberg, Germany).

### 4.4. Structural Characterization

#### 4.4.1. Density, Porosity, Microstructure

Uniform bone plugs were machined from distal femur metaphyseal cross sections, using the methods previously described. Bone plug volume was measured in cubic millimeters and converted into milliliters while dry weight was measured in milligrams. Microstructure measurements were derived from micro-CT imaging. Images were inverted to visualize porous domains and calculate average pore diameter. Porosity was calculated as percentage by subtracting specimen volume fraction from 1 [59]. Trabecular strut diameter, density, and spacing were measured and compared between bone and scaffold samples.

#### 4.4.2. Finite Element Analysis

DICOM files (Digital Imaging and Communications in Medicine) from bone and scaffold specimens were imported into Scanco analysis software for non-destructive biomechanical testing using voxel-based finite element analysis (FEA) [79]. Testing was performed on 15 mm^3^ sub-volumes of micro-CT specimens. Sample strength was defined by yield stress (von Mises stress). Because naturally-derived bone scaffolds undergo remodeling in the host until eventually being replaced, yield stress was of particular interest in biomechanical assessment [79,80].

#### 4.4.3. Mechanical Compression Testing

Bone (*n* = 12) and scaffold (*n* = 12) specimens from the same individual femurs were machined into uniform cylinders using a drill press-mounted diamond hole saw bit (Lenox, East Longmeadow, MA) and stored at −80 °C. Specimens were soaked in normal saline for two h at room temperature prior to mounting them between stainless steel platens for axial compression testing on a material testing machine (MTS) (Instron^®^, Norwood, MA, USA). Mounted specimens were preloaded to 5 N in a 2 kN load cell and compressed with a crosshead speed of 0.5 mm/s until fracture or 80% strain was reached [64,81,82]. Ultimate stress and strain were calculated from the first peak of the generated stress-strain curve (Bluehill^®^ Software, Instron, Norwood, MA, USA) while Young’s modulus was derived from the slope of the stress-strain curve’s linear portion.

### 4.5. Proteomics

#### Mass Spectrometry

The identities of proteins within the porcine bone scaffolds (*n* = 3) were analyzed using mass spectrometry (MS). For comparison against an analogous commercially available bone graft product, cancellous demineralized bone matrix (DBM) (Musculoskeletal Transplant Foundation, Edison, NJ, USA) samples (*n* = 3) were also analyzed. Specimens were cryomilled in liquid nitrogen, reduced with dithiothreitol, and alkylated with iodoacetamide before overnight digestion with trypsin (Trypsin Gold, Promega, Madison, WI, USA) at pH 8.0. Mass spectrometry analysis was performed using an electrospray ionization (ESI) ion trap LTQ Orbitrap XL hybrid FTMS (Fourier Transform Mass Spectrometer) (Thermo Fisher Scientific, Waltham, MA, USA) coupled to a nanoACQUITY UltraPerformance LC (UPLC) system (Waters, Milford, MA, USA). The Mascot version 2.2.07 (Matrix Science, Boston, MA, USA) search engine was used to search the protein sequence databases (Swiss Prot and National Center for Biotechnology Information (NCBI) NCBInr) with the following settings: MS/MS ion search, taxonomy filter = Sus scrofa (pig)/Homo sapiens (human) all entries, enzyme = trypsin, maximum missed cleavages = 1, fixed modifications = carbamidomethyl (C), variable modifications = deamidated (NQ)/formyl (N-term)/oxidation of Met and Pro (to detect hydroxyl proline in collagens). MS hits with protein scores ≥100 were reported [83,84]. Results were queried in ExPASy proteomics Bioinformatics Resource Protocol, as well as the UniProt Knowledgebase (UniProtKB) [40]. Functional information of queried protein sequences along with their referenced accession numbers was extracted from UniProtKB. Analysis of native donor porcine bone was deferred because protein density would preclude informative analysis.

### 4.6. Statistical Analysis

Values are reported as the mean ± standard error and compared with independent samples *t*-tests with α = 0.05 to determine significance.

## 5. Conclusions

Results presented in the current study show that our decellularization protocol can derive a scaffold from porcine bone that is devoid of donor cellular material while preserving the microarchitecture critical to osteoconduction, and potentially, the growth factors thought to contribute to the osteoinductive potential of bone graft substitutes.

## 6. Patents

Decellularization and oxidation protocol (U.S. Patent #8,221,777 B2).

## Figures and Tables

**Table 1:** Structural Characterization Indices.

Parameter	Donor Bone	Scaffold	
Density	1366 ± 20 mg/mL	570 ± 9 mg/mL	*p <* 0.01
Porosity	79.5 ± 9.1%	69.1 ± 11.1%	*p* = 0.2
Anisotropy	1.88 ± 0.1	1.65 ± 0.1	*p* = 0.1
Mean Pore Size	458.5 ± 66.3 µm	474.2 ± 76.2 µm	*p* = 0.8
Strut Thickness	142.8 ± 27.8 µm	121.7 ± 21.9 µm	*p* = 0.3

Donor bone was decellularized to create scaffolds. Density and ultrastructure parameters are presented as the mean ± standard error and compared by independent samples *t*-tests.

**Table 2:** Biomechanical Testing Indices.

	Donor Bone	Scaffold	
**FEA Modeling**			
Yield Stress (von Mises)	11,372 ± 286 MPa	10,922 ± 327 MPa	*p* = 0.39
Stiffness	31,921± 8250 N/mm	18,840 ± 6603 N/mm	*p* = 0.26
Failure Load	148.0 ± 35.7 MPa	89.5 ± 29.5 MPa	*p* = 0.25
**Mechanical Testing**			
Young’s Modulus	236.6 ± 11.8 MPa	114.2 ± 17.8 MPa	*p* < 0.01
Stiffness	1544.0 ± 76.2 N/mm	727.6 ± 120.1 N/mm	*p* < 0.01
Failure Load	14.5 ± 1.8 MPa	13.6 ± 1.8 MPa	*p* = 0.72
Strain at Failure	0.088 ± 0.006	0.230 ± 0.014	*p* < 0.01

Biomechanical properties of representative unprocessed bone and decellularized scaffolds are presented as the mean ± standard error and compared by independent samples *t*-tests. Parameters were measured by finite element analysis (FEA) modeling as well as mechanical compression testing on an Instron^®^ MTS.

**Table 3:** Mass Spectrometry Proteomics Analysis.

Protein Detected	UniProtKB #	Porcine Scaffold Samples	Human DBM Samples	Protein Function
Chondroadherin	F1RT93 O15335	3/3	3/3	Promotes the attachment of chondrocytes, fibroblasts, and osteoblasts. JAK-STAT cascade signaling
Collagen alpha-1(I) chain	P02452	3/3	3/3	Protease binding, metal ion binding, bone trabeculae formation, enchondral ossification, cell response to TGF β, collagen fibril organization, cell response to mechanical stimuli, and osteoblast differentiation
Collagen alpha-2(I) chain	A0A1S7J1Y9 P08123	3/3	3/3	Extracellular matrix structural constituent, SMAD signaling, collagen fibril organization, cytokine signaling, and TGF β receptor signaling
Pigment epithelium-derived factor	Q0PM28 P36955	3/3	3/3	Neurotrophic protein, inhibitor of angiogenesis, cell proliferation
Serum albumin	P08835 P02768	3/3	3/3	Main plasma protein
Alpha-2-HS-glycoprotein	P29700 P02765	3/3	2/3	Endopeptidase inhibitor; negative regulation of biomineral tissue development; negative regulation of bone mineralization, positive regulation of phagocytosis
Lumican; fibromodulin	Q9TTB4 P51884	3/3	2/3	Primary role in collagen fibrillogenesis, collagen binding, response to growth factor
Biglycan	Q9GKQ6 P21810	2/3	3/3	Involved in collagen fiber assembly, protein kinase inhibitor, negative regulation of JAK-STAT cascade, ECM structure, blood vessel remodeling, and cytokine signaling
Annexin A5	P08758	3/3	0/3	Blood coagulation
Hemoglobin subunit beta	P02067 P68871	2/3	0/3	Highly abundant blood protein, oxygen transport
Alpha-1-antiproteinase	P50447 P01009	2/3	0/3	Serine protease inhibitor; found in high levels in blood
Vitronectin	P48819 P04004	0/3	3/3	Cell adhesion and spreading factor in serum and tissues, cell proliferation, wound healing, and cell migration
Prothrombin	F1SIB1 P00734	0/3	2/3	Blood coagulation, converts fibrinogen to fibrin, activates coagulation factors

Mascot protein scores >100 (www.matrixscience.com) are listed with respective species-specific UniProtKB accession numbers. Three separate porcine scaffold and three human DBM samples were separately analyzed and the number of these samples recording hits for the listed proteins is reported. Proteins observed in only one sample or those which had protein scores <100 were excluded from analysis. Functional information of reported proteins was derived from the UniProtKB database [40].
